# Colorectal Cancer Metastasis to the Thymus Gland: Rare Presentation of Colorectal Cancer as Anterior Mediastinal Mass

**DOI:** 10.1155/2017/6581965

**Published:** 2017-01-02

**Authors:** H. Charles Peters, Xiuli Liu, Atif Iqbal, Lisa A. Cunningham, Sanda A. Tan

**Affiliations:** ^1^Department of Surgery, University of Florida College of Medicine, Gainesville, FL, USA; ^2^Department of Pathology, Immunology, and Experimental Pathology, University of Florida College of Medicine, Gainesville, FL, USA

## Abstract

Despite improved screening modalities, 15–25% of newly diagnosed colorectal cancers are metastatic at the time of diagnosis. The vast majority of these cases present as hepatic metastasis; however, 22% present with concomitant extrahepatic disease. The thymus gland is an uncommon site of metastasis for any primary malignancy, particularly, colorectal cancer given its vascular and lymphatic drainage. This case report details our experience with a rare case of colorectal cancer metastasis to the thymus gland presenting as a symptomatic mediastinal mass.

## 1. Introduction

Despite improved screening modalities, 15–25% of newly diagnosed colorectal cancers are metastatic at the time of diagnosis [1]. As the third most common cancer in the United States, this population represents a significant medical and financial challenge to our healthcare system [2]. The majority of patients who present with metastatic disease have hepatic lesions; although 22% present with extrahepatic disease only or with concomitant extrahepatic disease [3]. The most common sites of extrahepatic colorectal metastasis include the lungs (51%), lymph nodes (26%), and peritoneum (12%) with metastasis to ovary, bone, brain, adrenals, and locoregional recurrence (11%) contributing to a minority of extrahepatic disease burden [3]. The treatment of patients with extrahepatic disease remains controversial with no clearly defined treatment algorithm though studies have demonstrated the efficacy of systemic therapy along with surgery rendering patients with no evidence of disease.

The thymus gland is a rare site of metastasis for any primary malignancy, particularly, colorectal cancer given its vascular and lymphatic drainage [4]. To our knowledge, only one case of colorectal adenocarcinoma metastasizing to the thymus gland has been documented, by Lee et al. in 2015 [5]. This case report details our experience with a rare case of colorectal metastasis to the thymus gland in a young, healthy male. We focus on immunophenotypic and molecular characteristics of this lesion along with the presence of a possible metastasis via lymphovascular involvement to the duodenum.

## 2. Case Report

**Figure 1:**
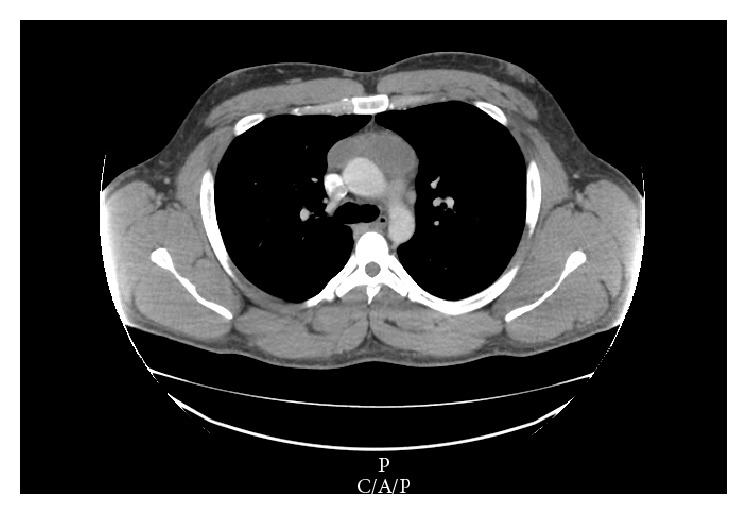
Computed tomography scan showing a 7.6 × 2.6 cm anterior mediastinal mass.

A 35-year-old male with a past medical history only significant for hypertension and a family history significant for inflammatory bowel disease (IBD) and colon cancer in a maternal grandmother presented to the emergency department with complaints of chest pain, shortness of breath, and unintentional 30 pound weight loss for 3 months. Workup identified a 7.6 × 2.6 cm anterior mediastinal mass with associated lymphadenopathy seen on CT imaging with no lesions noted elsewhere (Figure 1). Differential diagnosis for the anterior mediastinal mass consisted of both benign etiologies such as thymoma or thymic cyst and malignant etiologies such as lymphoma, primary germ cell tumor, or a metastatic lesion.

The patient underwent a robotically assisted thymectomy via a left thoracoscopic approach by Cardiothoracic Surgery. The mediastinal mass was noted to involve the thymus under thoracoscopy and was partially resected due to extension into the right thorax. Pathology from the lesion revealed a poorly differentiated adenocarcinoma with enteric histomorphology and >5 positive lymph nodes demonstrating extranodal extension with prominent lymphovascular invasion. No residual thymic tissue was identified. The pathologists subjected the specimen to various immunophenotypic stains (Table 1). The adenocarcinoma of the anterior mediastinum was diffusely positive for CDX2, CK20, and *β*-catenin in addition to CK7 positivity. The tumor was negative for TTF-1, PAX8, cKIT, and SALL4, markers for lung adenocarcinoma, thyroid carcinoma, thymic carcinoma, and germ cell tumor, as would be expected from thymic tissue. We were unable to definitively identify whether the mass represented a rare primary thymic adenocarcinoma arising within a thymic cyst or a metastatic lesion giving this clinical presentation and the young age of the patient. The anterior mediastinal tumor was microsatellite stable (MSS) on microsatellite instability testing. Genetic mutation analysis by next generation (Ion Torrent) sequencing on formalin-fixed paraffin-embedded tumor tissue (Department of Anatomical Pathology, University of Florida, Gainesville) revealed that the adenocarcinoma in the anterior mediastinum harbored mutations in KRAS (c.38G>A), SMAD4 (c.1081C>T), and MET (c.2962C>T). A consensus was achieved that this was probably a metastatic lesion, likely colonic in origin, given the aberrant nuclear expression of *β*-catenin frequently seen in colonic-derived adenocarcinomas.

**Figure 2:**
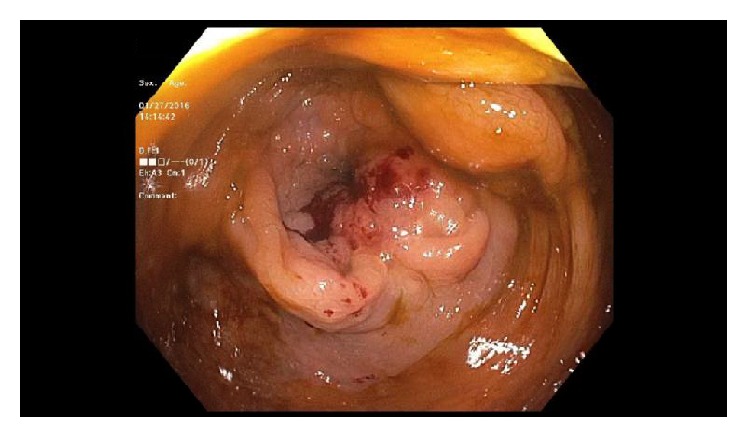
Colonoscopic image of the cecal mass.

A search for a potential occult primary malignancy was conducted with a positron emission tomography scan (PET/CT) along with upper and lower endoscopy. PET scan revealed a residual fluorodeoxyglucose (FDG) avid lesion in the anterior mediastinum and a lesion in the right colon near the hepatic flexure. Endoscopy identified a small, nonbleeding, ulcerated lesion in the duodenum as well as a large, nonobstructing ascending colon mass (Figure 2). Biopsies confirmed a primary colonic malignancy and a focus of metastatic adenocarcinoma limited to the lymphovascular channels in the mucosal biopsy taken from the duodenum. No precursor lesion (i.e., adenoma) was identified in the duodenal biopsy and the consensus was that this was likely a lymphovascular metastatic deposit from the colonic primary tumor. Carcinoembryonic antigen (CEA) was within normal limits at 1.3 ng/mL.

**Figure 3:**
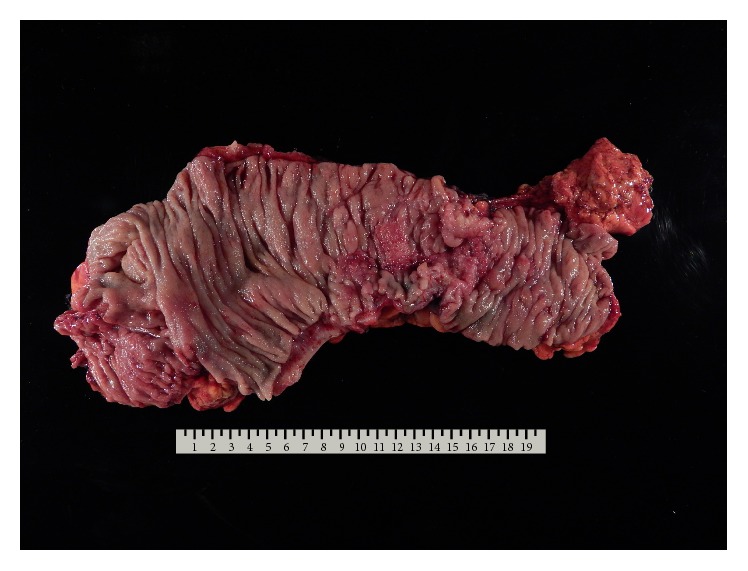
Pathologic specimen after surgical resection for the cecal mass.

The case was presented at a multidisciplinary tumor board and a consensus was developed to proceed with resection of the primary colonic malignancy followed by systemic chemotherapy. The patient underwent an uncomplicated laparoscopic right hemicolectomy (Figure 3) by Colorectal Surgery with an uneventful recovery. Final pathology of the colonic specimen revealed an invasive, poorly differentiated, adenocarcinoma arising from a tubular adenoma with high grade dysplasia and thirteen of twenty-eight lymph nodes positive for metastatic adenocarcinoma. Despite aggressive en bloc resection of the pericolonic tissue the pathology showed a positive radial margin giving a final pathologic stage of pT4apN2bpM1. Morphologically and genetically, the colonic adenocarcinoma was identical to the resected adenocarcinoma in the anterior mediastinum (Table 1). The overall prognosis for the patient is poor and given the final pathologic and genetic test results, the patient is going to continue on palliative chemotherapy with FOLFOX-bevacizumab.

## 3. Discussion

Extrahepatic colorectal metastasis most commonly involves the lungs, lymph nodes, and peritoneum and represents a significant surgical and oncologic challenge. This case is unique in that it involves colonic cancer metastasis to the thymus gland, a rare site of metastasis for any malignancy, although cases of metastatic breast, thyroid, testicular, and prostate cancer have been documented. The histologic architecture of the thymus has been theorized to play a large role in its immunity from metastatic disease. The thymus gland's role in cellular immunity and the presence of a blood-thymus barrier typically prevents the parenchyma of the thymus from direct contact with tumor cells or antigens [6]. However, the blood vessels, lymphatics, and nerves making up the capsule and interlobular septum of the thymus gland provide the most plausible route for seeding of tumor cells from primary tumors [7]. Ours is the first case report to show metastatic colorectal cancer to the thymus with immunophenotypic and genetic comparisons of the primary tumor and the thymic metastasis. Taking all clinicopathological and molecular information, the final diagnosis of this case was colonic adenocarcinoma with extensive lymphatic permeation in the colon with metastasis to the anterior mediastinum including the thymus and possibly the duodenum.

Optimal management of these rare patients with thymic metastasis of colorectal cancer is not known. Traditionally, the presence of extrahepatic colorectal metastasis has been a contraindication to attempts at curative surgical resection but several studies have demonstrated a survival benefit with aggressive resection of all colorectal metastasis, particularly pulmonary metastasis [8]. Even if not completely resectable, the effects of cytoreductive surgery in reducing tumor burden and associated immunosuppressive effects of tumor cells thus allowing the patient's natural immunological defenses and chemotherapy to be more effective continue to be controversial [9]. The paucity of cases involving colorectal metastasis to the thymus gland and duodenum makes evidence-based treatment recommendations impossible. Treatment decisions should be individualized with input from a multidisciplinary tumor board. Applying data from pulmonary colorectal metastasis to thymic metastasis, an attempt to render the patient free of obvious measurable disease should be made, if possible.

## Figures and Tables

**Table 1:** Morphology and immunophenotype of the resected colonic and mediastinal adenocarcinomas.

Features	Colonic adenocarcinoma	Anterior mediastinal adenocarcinoma
Morphology		
Precursor lesion	Yes	No
Differentiation	Poor	Poor
Micropapillary feature	Present	Present
Lymphovascular invasions	Extensive	Extensive
Lymph node metastasis	Present	Present
Immunophenotype		
CDX2	Diffuse and strong immunoreactivity	Diffuse and strong immunoreactivity
CK20	Diffuse and strong immunoreactivity	Diffuse and strong immunoreactivity
CK7	Positive	Positive
*β*-catenin	Cytoplasmic and nuclear staining	Cytoplasmic and nuclear staining
TTF-1	N/A	Negative
PAX 8	N/A	Negative
cKIT	N/A	Negative
SALL4	N/A	Negative
Microsatellite status		
Microsatellite stable	Yes	Yes

N/A: not performed.
